# Genome-Based Analyses of Fitness Effects and Compensatory Changes Associated with Acquisition of *bla*_CMY_-, *bla*_CTX-M_-, and *bla*_OXA-48/VIM-1_-Containing Plasmids in *Escherichia coli*

**DOI:** 10.3390/antibiotics10010090

**Published:** 2021-01-19

**Authors:** Michael Pietsch, Yvonne Pfeifer, Stephan Fuchs, Guido Werner

**Affiliations:** 1 Robert Koch Institute, Department Infectious Diseases, Division Nosocomial Pathogens and Antimicrobial Resistances, Wernigerode Branch, 38855 Wernigerode, Germany; pietschm@rki.de (M.P.); pfeifery@rki.de (Y.P.); 2 Robert Koch Institute, Department Methodology and Research Infrastructure, Division Bioinformatics, 13353 Berlin, Germany; fuchss@rki.de

**Keywords:** AmpC, ESBL, carbapenemase, fitness burden, compensatory mutation

## Abstract

(1) Background: Resistance plasmids are under selective conditions beneficial for the bacterial host, but in the absence of selective pressure, this carriage may cause fitness costs. Compensation of this fitness burden is important to obtain competitive ability under antibiotic-free conditions. In this study, we investigated fitness effects after a conjugative transfer of plasmids containing various beta-lactamase genes transferred into *Escherichia coli*. (2) Methods: Fourteen beta-lactamase-encoding plasmids were transferred from clinical donor strains to *E. coli* J53. Growth rates were compared for all transconjugants and the recipient. Selected transconjugants were challenged in long-term growth experiments. Growth rates were assessed at different time points during growth for 500 generations. Whole-genome sequencing (WGS) of initial and evolved transconjugants was determined. Results: Most plasmid acquisitions resulted in growth differences, ranging from −4.5% to 7.2%. Transfer of a single *bla*_CMY-16_-carrying plasmid resulted in a growth burden and a growth benefit in independent mating. Long-term growth led to a compensation of fitness burdens and benefits. Analyzing WGS revealed genomic changes caused by Single Nucleotide Polymorphisms (SNPs) and insertion sequences over time. Conclusions: Fitness effects associated with plasmid acquisitions were variable. Potential compensatory mutations identified in transconjugants’ genomes after 500 generations give interesting insights into aspects of plasmid–host adaptations.

## 1. Introduction

Microbial antibiotic resistance is a major challenge to public health. Resistance to most therapeutically important agents is increasing worldwide [1]. A generally discussed countermeasure to prevent and revert an increasing resistance development is to use antibiotics properly, thus reducing the pressure to give resistant bacteria a selective advantage over the susceptible population. In line with this argument, the question of a potential fitness burden coming along with resistance acquisition in bacteria is central. It is mostly set as a prerequisite that susceptible bacteria are fitter than their multidrug-resistant counterparts and thus, have an evolutionary advantage if selection pressure by antibiotic use is released. Consequently, reduction of antibiotic use (reduced pressure) should immediately or with a certain delay lead to decreasing resistance rates and frequencies.

Mutation-based resistance such as ciprofloxacin resistance in enterobacteria or rifampicin resistance in mycobacteria and their effects on bacterial fitness and fitness compensation have been studied in greater detail, and corresponding results mainly questioned the textbook knowledge about resistance acquisition and fitness burdens [2,3,4]. If a fitness burden was measurable at all, bacteria frequently and quickly “adapted” to these challenges by selecting for populations demonstrating compensatory changes leading to fitness compensations [3,4,5,6,7,8]. Whereas the effect has been described, its genetic backgrounds are much less understood. A lot of clinically important and trans-sectoral resistance problems come along with acquired resistance traits, like beta-lactamases, which are mainly located on mobile genetic elements, such as Integrative and Conjugative Elements (ICE) including plasmids, and which are horizontally acquired. This situation even complicates the above-described setting since fitness is influenced by resistance and plasmid acquisitions and since horizontal gene transfer itself may come along with an influence on fitness [9,10]. Many of these interacting factors and conditions were collated and reviewed recently [11].

Resistance to beta-lactams in Enterobacteriaceae like *Escherichia coli*, *Klebsiella pneumoniae, Enterobacter cloaceae* and *Citrobacter freundii* is mainly mediated by acquired resistance properties associated with the presence of beta-lactamases, including extended-spectrum-β-lactamases (ESBLs), plasmid-mediated AmpC beta-lactamases (pAmpCs) and carbapenemases [12,13,14]. The corresponding genes are mainly plasmid-located or reside on chromosomal ICEs. In some Enterobacterales, AmpC-type lactamase genes are genus- and/or species-specific, but their expression is low controlled by a tightly regulated repressor. Promotor and repressor mutations could lead to a derepressed AmpC expression, for instance in *Enterobacter* and *Citrobacter*, resulting in third generation cephalosporin resistance. However, this resistance feature is not transferable and thus has not received greater Public or One Health attention. The success to disseminate β-lactaman resistance widely among the different sectors is linked to distinct mobile vectors and/or bacterial lineages. Specific “beta-lactamase gene/plasmid” and “beta-lactamase gene/plasmid/strain” combinations successfully disseminated in single sectors but are almost absent among others. Exemplarily, *E. coli* isolates of sequence type ST131 carrying a *bla*_CTX-M-15_ ESBL gene successfully spread among healthy humans and human patients worldwide, but are much less frequently encountered in wild animals and livestock [15]. In contrast, AmpC-type lactamases like CMY-2 are prevalent among *E. coli* from livestock, especially chicken and poultry, but their prevalence varies among humans, with higher rates in Asia and comparably low rates in Europe [16,17,18]. Several German studies assessed the prevalence of ESBL- and AmpC-mediated resistance among *E. coli* in food animals, food and humans [19,20,21,22]. As part of this interdisciplinary research initiative, we demonstrated a wider prevalence of *bla*_CMY-2_ as the most prevalent pAmpC-type beta-lactamase gene among livestock and food, but less frequently among humans in Germany [23]. The *bla*_CMY-2_ gene was preferably located on plasmids of IncK and IncI types. 

The preferred combination of certain “beta-lactamase gene/plasmid” or “beta-lactamase gene/plasmid/strain” combinations potentially has a fitness background. We hypothesized that such fitness effects may be measurable with a comparable simple experimental setting used in the present study. If plasmids would be adapted to their bacterial host, then transferring them to a novel host should result in measurable fitness costs. We selected 47 donor strains of *E. coli*, *K. pneumoniae, K. oxytoca* and *E. cloaceae* possessing various pAmpC (*bla*_CMY-2/16_), ESBL (*bla*_CTX-M-1/-14/-15_, *bla*_SHV-12_) and carbapenemase (*bla*_OXA-48_, *bla*_VIM-1_, *bla*_NDM-1_) genes. The resistance genes were transferred by broth mating into the *E. coli* J53 recipient. Transconjugants demonstrating highest growth differences compared to the recipient were selected for subsequent long-term growth experiments. Growth rates were assessed at different time points at the beginning, during and at the end of the long-term cultivation. Whole-genome sequences of initial and evolved transconjugants were compared to determine compensatory mutations to ameliorate initial fitness effects.

## 2. Results

### 2.1. Transfer Experiments

Altogether, 43 strains of *E. coli* (n = 38), *K. pneumoniae* (n = 3), *K. oxytoca* (n = 1) and *E. cloacae* (n = 1) were selected as donors for mating experiments, with *E. coli* J53 as a recipient (see Materials Section and Supplementary Table S1). Of these, fourteen donors transferred beta-lactamase-mediated resistance into *E. coli* J53 (Table 1). The transferred plasmids varied in size from 60 to 250 kb and belonged to different incompatibility groups. All transconjugants contained at least one ESBL, AmpC or carbapenemase gene, and three transconjugants additionally harbored the beta-lactamase gene *bla*_TEM-1_.

**Figure 1:**
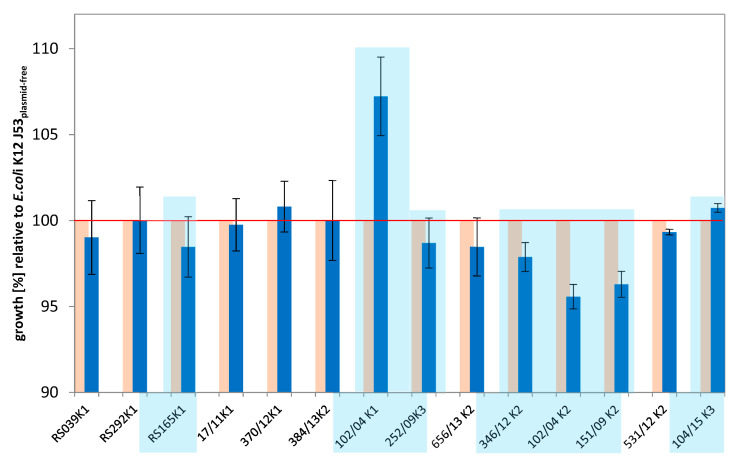
Growth rates of the transconjugants (blue) relative to the growth rate of the plasmid-free recipient E. coli J53 Azir (yellow). Isolates se-lected for further analysis are highlighted by a light blue background.

The initial relative fitness load of the transconjugants after plasmid uptake compared to the plasmid-free recipient strain was determined by growth experiments. The deviations of the relative fitness of the transconjugants to the plasmid-free recipient ranged from approximately −5% to +7%. For most transconjugants, the deviations ranged from 1–2% (Figure 1). Isolates with higher negative deviation in relative growth rate were 346/12 K2, 102/04 K2 and 151/09 K2. One isolate, 102/04 K1, showed a higher fitness compared to recipient *E. coli* J53. 

In addition to the growth rate, differences in growth behavior were observed. Outside the exponential growth phase, which was used as a marker for the relative fitness calculation, different curve progressions in the late log or stationary phase were observed (Supplementary Figure S1). Strains with the highest growth rates did not always show the highest endpoint optical density (OD) (see 102/04 K1). However, no significant delay in the lag phase was observed. Simplified, it was assumed that with continuous and competitive growth, a higher growth rate would outcompete other strains over time and lead to a fitness advantage and thus to higher abundance over time. Therefore, the growth rate in the exponential growth phase was used as a marker for the fitness of the isolates.

For in-depth analyses, seven of the transconjugants (102/04 K1, 102/04 K2, RS165 K1, 252/09 K3, 346/12 K2, 151/09 K2 and 104/15 K3), expressing the most significant changes in growth, were selected for further experiments (highlighted in Figure 1).

### 2.2. Long-Term Growth Experiments

To identify potential fitness compensation effects, long-term growth experiments over 500 generations of the seven selected transconjugants were performed. The initially determined factor for the increase in generations per passage was 8.96. To achieve generations G_500_, the cultures of the transconjugants were therefore inoculated consecutively for 56 days and sampled and analyzed at generations G_200_ and G_500_ (Supplementary Table S2). 

Since it was assumed that within the long-term cultures, a population diversity caused by different subpopulations could develop, four randomly selected colonies of each plated long-term culture were selected and analyzed.

**Figure 2:**
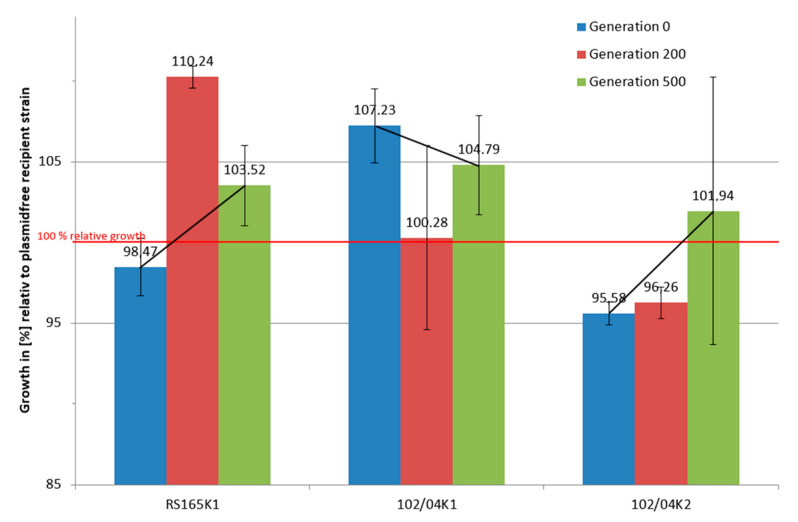
Development of the relative fitness of transconjugants 102/04 K1, 102/04 K2 and RS165 K1 compared to the recipient during long-term cultivation for 500 generations. Aggregated values at the start point (G_0_), after 200 generations (G_200_) and 500 generations (G_500_) are shown. Error bars depict standard deviations from three independent experiments (mean values from single experiments based on technical triplicates).

In general, all isolates showed a rather constant or strongly negative trend (102/04 K1 and 346/12 K2) of the relative fitness for generation G_500_ compared to G_0_. Interestingly, the relative fitness values determined for generation G_200_ were in some cases far above (RS165K1, +9.2%) or far below (151/09 K2, −9.0%) the fitness mean of G_0_ to G_500_ (Figure 2). The presence of beta-lactamase genes was tested positive by PCR in all selected long-term cultivated transconjugants in each generation. A change in the respective plasmid-mediated antimicrobial resistances was not observed, and corresponding resistance patterns remained stable over time (Supplementary Table S3).

### 2.3. Genome Reconstruction and Genome Comparisons

After 500 generations of long-term cultivation, three transconjugants with plasmids carrying *bla*_CTX-M-14_ (RS165 K1) and *bla*_CMY-16_ (102/04 K1 and 102/04 K2) were selected for whole-genome comparison studies. 102/04 K1 and 102/04 K2 were chosen because plasmid acquisition in G_0_ transconjugants demonstrated opposite fitness effects and RS165 K1 because the G_200_ value showed a remarkable fitness gain (Figure 2). Generation G_0_ isolates of the transconjugants 102/04 K1 and 102/04 K2 were sequenced using PacBio technology to create a reference sequence. 102/04 K1/K2 isolates of generation G_0_ and one evolved transconjugant 102/04 K1 G_500_ were sequenced using Illumina MiSeq. RS165 K1 G_0_ and three evolved transconjugants of different generations of RS165 K1 (G_200_-1, G_200_-3, G5_00_-3) were sequenced using Illumina MiSeq. Whole-genome sequence analyses enabled the detection of base substitutions, deletions, insertions or modifications of other kinds in the chromosome or plasmids in the different isolates. Identified sequence modifications were examined for their possible influence on the fitness of the isolates.

SMRT sequencing and subsequent HGAP assembly of 102/04 K1 and K2 resulted in two ring-closed sequences for each isolate. A *blastn* analysis confirmed and assigned these contigs to the expected chromosome and plasmid. The reconstruction of the genome sequences of the isolates of the latter generation based on Illumina reads was performed using the reconstructed sequences as a reference (Supplementary Tables S4 and S5).

**Figure 3:**
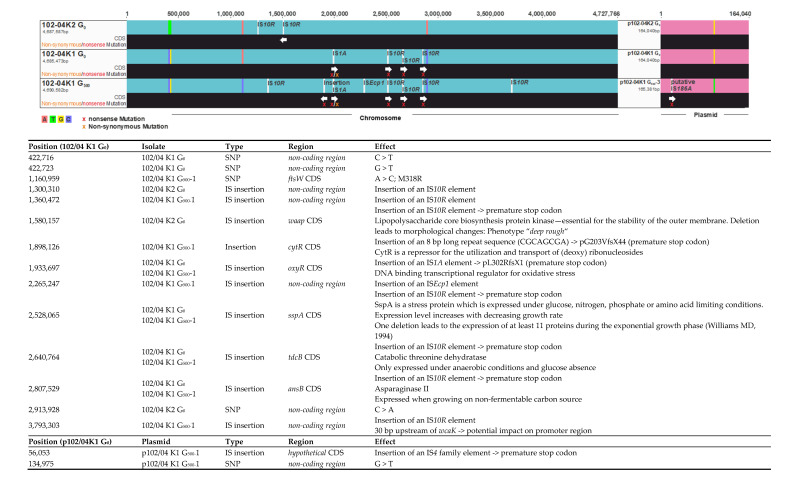
Schematic presentation of the genetic changes of transconjugant isolates 102/04 K1 und K2 after long-term growth. Mutations in the chromosomes and amino acid changes are marked at the respective positions. Base pair substitutions are marked by color. Positions of modified IS elements are highlighted by white bars. White arrows point towards changes in coding sequences.

**Figure 4:**
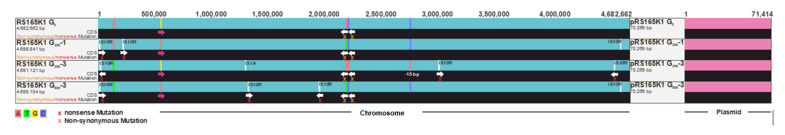

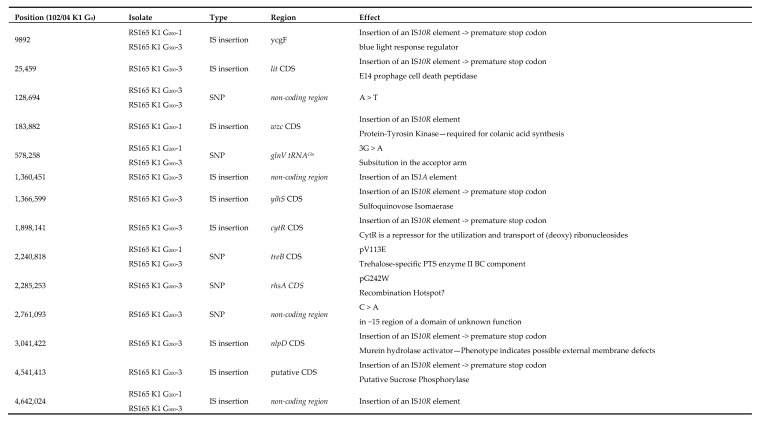
Schematic presentation of the genetic changes of transconjugant isolates RS165 K1 after long-term growth. Mutations in the chromosomes and amino acid changes are marked at the respective positions. Base pair substitutions are marked by color. Positions of modified IS elements are highlighted by white bars. White arrows point towards changes in coding sequences.

The reconstructed genome and plasmid sequences of the isolates of the different generations of RS165 K1, and the three 102/04 transconjugants, were examined for differences. Apart from a few SNPs and insertion sequences, the genome and plasmid sequences of the different transconjugants 102/04 K1, K2 and RS165 K1 were homologous to each other and did not show any genomic rearrangements (Figure 3 and Figure 4).

Comparison of the three 102/04 transconjugant genomes showed a total of four chromosomal and one plasmidic SNP. In addition, eleven different insertion sites were found (ten on the chromosome and one on the plasmid sequence). Of these changes in the nucleotide sequence, eight were located in non-coding regions. One SNP caused a non-synonymous mutation of a protein sequence and seven insertion sequences led to nonsense mutations of the encoded amino acid sequences due to premature stop codons. Detailed information on the individual nucleotide sequence changes is shown in Figure 3. Most SNPs and insertion sites were observed between isolates 102/04 K1 and 102/04 K2, while isolates 102/04K1 G_0_ and G_500_, and 102/04 K2 G_0_ and G_500_, were nearly identical in their SNPs and insertion sites (Figure 3). However, seven SNPs or differences caused by insertions were also observed between these follow-up isolates. The inserted insertions’ sequence (IS) elements belonged to the element IS*10R* in almost all cases.

Comparison of the four RS165 K1 isolates revealed 14 changes in the chromosome (Figure 4). The plasmid sequences did not show any differences. The chromosomal changes related to five SNPs, two of which were located in noncoding regions and two of which led to non-synonymous mutations. The remaining SNP was located at position 3 (acceptor arm) of the *tRNA*^Gln^ (*glnV*) in isolate RS165K1 G_200-1_. Furthermore, nine IS element insertions were observed, seven of which were localized in coding sequences and led to non-synonymous mutations. Generation G_200_ isolates showed different mutations among each other. In the generation G_500_ isolate, identical mutations as in the G_200_ isolate were detected (Figure 4). As in transconjugants 102/04 K1/K2, the majority of the differences in the nucleotide sequences were due to the insertion of IS*10R* at different positions in the chromosome.

## 3. Discussion

### 3.1. Comparative Growth Experiments

Measuring bacterial growth under optimal conditions regarding temperature, aeration, rich nutrition conditions and without a competing flora is a quite artificial setting to determine bacterial fitness and for sure not the best model to mimic the natural habitat of a given bacterium. Consequently, competitive growth experiments to measure fitness effects should be carried out under the most practical conditions possible, meaning either in vitro or even better, performed under in vivo conditions of an infection [24,25]. Nevertheless, measuring growth dynamics and comparing plasmid-free and plasmid-containing isogenic strains allows a first and deep look into the complex interplay of plasmid (resistance gene)–host interactions. 

The horizontal spread of resistance genes has a high impact on the dissemination of a number of resistance properties of high public health impact. For Gram-negative bacteria resistance to third generation cephalosporins mediated by ESBLs and pAmpC enzymes, resistance to carbapenems mediated by carbapenemases and resistance to colistin mediated by Mcr variants are of major importance. These resistance genes are horizontally acquired and spread across bacterial genera and sectors [20,23,26,27]. The experimental setting presented here does not allow differentiating between fitness effects associated with plasmid and/or resistance gene acquisition. We consider this as a rather theoretical problem since many of the analyzed resistance genes included in this study have a strong association with a corresponding ICE or plasmid background and thus, resistance gene acquisition in nature is mainly and directly associated with acquisition of a corresponding plasmid. In this regard, the experimental setup always measured the combined effect of a plasmid and pAmpC, ESBL or carbapenemase gene acquisition.

Performing competitive growth experiments with progenitor and descendent strain variants in the same experimental setting, such as a combination of recipient and transconjugant cells, allow determining an immediate and interconnected fitness effect and measuring much lower fitness effects [28,29,30]. However, in a setting as described here, where conjugative plasmid transfer appears in liquid broth to a substantial amount, measuring growth rates in competitive experiments will always assess effects derived from de novo plasmid transfer and transconjugant propagation as well. Thus, we decided to perform, determine and evaluate growth in single-growth experiments only. 

As mentioned before, a simple comparative growth assay under laboratory conditions in rich medium was chosen in the present study to determine relative fitness. The fitness data of the generated transconjugants showed that the acquisition of a beta-lactamase gene-carrying plasmid did not necessarily have an effect on the fitness of the recipient *E. coli* J53. Only in about half of the transconjugants were negative growth effects, and in one case even a positive growth effect, observed (Figure 1). A correlation between plasmid size or replicon type on fitness could not be observed; due to the heterogeneous plasmid or transconjugants’ collection, it was difficult to conclude that a fitness effect derived from these parameters. 

The plasmid size can, however, influence the growth behavior of *E. coli,* as it was described in a study by Smith et al. [31]. The authors observed in isogenic clones that the presence of larger plasmids resulted in a longer lag phase than that of smaller plasmids. A fitness effect of this kind was not demonstrated in the present study. Nevertheless, the observation of Smith et al. shows that, in addition to the growth rate, other factors must be considered for a comprehensive fitness characterization [32]. As described earlier, plasmid stability and fitness derived from deeper and detailed analyses of variable vectors constructed and transferred into an *E. coli* background showed an effect of the plasmid size on fitness [31]. These and similar studies hide the fact that the analyzed system with an artificial vector, that is not naturally hosted in *E. coli,* largely differs from a tight and evolutionary fine-tuned plasmid–host link, which may be associated with much lower or no fitness costs. For instance, the transfer of a widely disseminated R1 plasmid (IncFII type) into an *E. coli* recipient did not reduce fitness of the transconjugants relative to the recipient. Long-term growth experiments were even capable of demonstrating a fitness advantage over time [33]. Schaufler et al. [34] were capable of curing *bla*_CTX-M-15_-containing plasmids from epidemic *E. coli* strains, hence giving the opportunity to investigate fitness effects of the tightly regulated interconnection between a plasmid and its host after loss of the corresponding plasmid. According to these and subsequent analyses [35], carriage of ESBL gene-containing plasmids did not come along with a fitness burden to the *E. coli* host, but rather with a fitness gain. Moreover, carriage of ESBL gene-carrying plasmids also increased virulence in the corresponding host. The association between acquisition of resistance and increased virulence partly mediated via multicomponent genetic elements is a general feature of larger ICEs, including plasmids [36,37].

Di Luca et al. [29] analyzed fitness costs associated with carbapenemase gene-carrying plasmids from *K. pneumoniae* (pG12-KPC-2; pG06-VIM-1) transformed into four *E. coli* recipients of various clonal lineages (ST10, ST69, ST95, ST537) and phylogenetic groups, such as A, B2 and D. Relative fitness costs were generally minor and in the range of 1.1% to 3.6% when compared between recipients and transformants. Fitness costs were dependent on the corresponding plasmid and the clonal background. 

The manifestation of a fitness burden after plasmid uptake in only a part of the transconjugants of the present study contradicts the assumption that the acquisition of a plasmid should always cause an initial negative fitness effect due to the additional burden of plasmid replication [32]. Based on a systematic review in which fitness costs due to acquired antimicrobial resistances were investigated, the authors Vogwill and MacLean were able to show that although the uptake of a plasmid is often associated with fitness costs, there were also very little or no fitness costs in the studies investigated and a correlation between plasmid size and fitness load was not sufficiently proven [38]. The authors concluded that regular plasmid uptake and loss in combination with corresponding adaptation processes occurred in the evolutionary past of host organisms. Therefore, new plasmid uptake does not necessarily lead to a loss of fitness, but only if new plasmid–host combinations are created or the plasmids acquire new, non-adapted genetic fragments, for instance, by horizontal gene transfer. This hypothesis is supported by the study of historical isolates from the Murray Collection, which showed that the “plasmid population” of isolates from the pre-antimicrobial period did not have a fundamentally different composition [39]. The absence of fitness costs in half of the investigated transconjugants in our study could be explained by a previously acquired plasmid–host adaptation. However, the observation of such different fitness values after uptake of plasmid p102/04 by *E. coli* J53 (K1: 107.23%, K2: 95.58% fitness compared to plasmid-free *E. coli* recipient) implies that further genetic effects have or could have a far greater influence on fitness than plasmid–host adaptations.

In a previous study using the Gram-positive enterococci as model organisms, it was shown that newly acquired ICEs including plasmids imposed an immediate biological cost in an *Enterococcus faecium* recipient [40]. However, the initial costs were mitigated through long term growth over 400 generations and beneficial plasmid–host associations rapidly emerged. It was also demonstrated that the beneficial genetic changes were imprinted either on the plasmid or on the chromosome. Second-round transconjugants either received the evolved resistance plasmid and thus, did not show a different growth behavior, or demonstrated again an initial fitness burden, suggesting that compensatory mutations were accumulated in the first-round transconjugants’ chromosomes [40].

### 3.2. Genome Comparisons

AmpC-mediated fitness costs were mainly addressed in the context of mutation-based genetic changes leading to derepressed chromosomal AmpC genes which mediate extended beta-lactam resistances [41,42]. This is the first study, we are aware of, which analyzed fitness effects of transferable *bla*_CMY_ genes and plasmids, respectively. Remarkably, we achieved opposite effects when we repeated the same experiment several times: transfer of the same CMY-2-containing plasmid into *E. coli* K12 J53 resulted either in a fitness burden or a fitness gain, suggesting that other as yet unknown circumstances may influence these biological consequences (see next section).

The observed diametric fitness effects between the transconjugants 102/04 K1 and 102/04 K2 was with 11.6% difference in relative growth, surprisingly and unusually large. Since mutations in the transferred plasmids of the two isolates could be excluded on the basis of the whole-genome data, it can be assumed that the different growth behavior of the strains was caused by chromosomal mutations. Between transconjugants 102/04 K1 and 102/04 K2, eight sequence differences caused by three SNPs and five IS elements at different positions were observed. Of these, three SNPs and three IS elements occurred in non-coding regions but could still have an impact on the gene expression. Interestingly, 102/04 K1 had a *sspA* gene truncated by one IS element. SspA (Stringent starvation protein A) is a stress protein expressed under glucose, nitrogen, phosphate or amino acid limiting conditions [43,44]. Several studies suggest that *sspA* is important for the stress response during the stationary phase and under nutrient-limited conditions in *E. coli*. The expression of *sspA* is positively regulated by the presence of *relA* and also increases with decreasing growth rate [43]. The exact role of SspA in transcription and cell physiology is still unclear. Williams et al. were able to show that a deletion of *sspA* during the exponential growth phase led to an altered expression of at least 11 proteins [43]. In addition, *sspA* mutants were found to be less fit than the wildtype under longer nutrient-limiting conditions or long stationary phases [43]. Hansen et al. could also show that *sspA* is necessary for the regulation of acid tolerance in *E. coli* [44]. Acid tolerance is an important ability of intestinal bacteria to survive the low pH during gastric passage to the intestinal tract [45]. Therefore, the loss of *sspA* may give rise to a presumed fitness advantage under artificial growth conditions, but under natural conditions (e.g., in the human host), the loss of *sspA* can be expected to adversely affect the fitness (or survivability) of *E. coli*.

In addition to *sspA*, *oxyR* was also found truncated in 102/04 K1. OxyR is a DNA-binding transcriptional regulator for oxidative stress and is indirectly involved in the regulation of more than 40 different gene products through its influence on mRNA stability [46].

### 3.3. Long-Term Growth Analysis

During long-term cultivation, a decreasing trend in the relative fitness of the strains was evident from generations G_200_ to G_500_. This is particularly surprising considering the fact that, according to observations in Richard Lenski’s Long-term Experimental Evolution Project (LTEE), a permanent increase in the relative fitness of the isolates could be observed over 60,000 generations [47]. However, since a slight decrease in relative fitness was also observed in the passaged plasmid-free recipient strain *E. coli* J53 Azi^r^ compared to generation G_0_, it can be assumed that other fitness-determining effects, which cannot be covered by the experimental approach used to measure fitness, affected the populations of the long-term cultures in the course of the experimental evolution. The discrepancy between expected fitness increase and observed fitness loss could probably have been better represented by direct competitive growth experiments. In addition, the process of daily inoculating new media represents a major hurdle for less competitive clones. By inoculating low-volume culture, clones with fitness factors other than a high growth rate, e.g., a higher final OD, may be selected. Over this effect probably led to the displacement of the expectedly fitter clones. The observation that a high endpoint OD often does not occur in isolates with the highest growth rates supports this assumption.

The different growth behavior and genetic differences in isolates of one generation implied that different sub-lineages from the original clone of generation G_0_ developed. The mutations leading to these descendants established in the population could not be investigated due to the small sample size of the sequenced isolates. However, the detection of identical SNPs and insertion elements in RS165 K1 G_200_ and G_500_ indicated that the subclone RS165 K1 G_200_ had established itself in the population. Whole-genome sequencing of further isolates or the entire population could have provided further information on the composition of the population.

The adaption of cells to the acquired plasmids and the growth conditions could be deduced from the growth behavior of the individual transconjugants. In previous studies, it was shown that under selective conditions, an adaptive evolution to the resistance gene-bearing plasmids occurred, which compensated for the fitness costs of acquiring plasmids [48,49,50]. Regulatory adaptive changes during co-evolution experiments occurred mostly in the bacterial host chromosome and not in the plasmids [48,51,52,53]. The occurrence of almost all mutations in the bacterial host chromosome of the evolved transconjugants in the present study indicates towards chromosomal regulatory adaptations. Excluding the highly mobile IS elements, a maximum of 2–4 SNPs occurred in 500 generations of evolved isolates. In the comparable LTEE, 45 SNPs were observed during the first 20,000 generations, which did not occur linearly but were more abundant in the early generations [47]. Whether and which SNPs or inserted IS elements have an effect on gene regulation and thus, on the fitness of the transconjugants of the present study, was not conclusively clarified. Studies by Harrison et al. and San Millan et al. have shown that fitness compensation can basically be caused by regulatory effects and copy number reduction of plasmids [52,53]. In contrast, Porse et al. observed that positive adaptations were achieved by deletions of large plasmid regions [54], which could be excluded in our study.

### 3.4. Limitations of the Study

The study has several limitations. First, various experimental settings exist to measure bacterial fitness costs and we are well aware that this model applied here does not reflect the situation multidrug-resistant pathogens encounter in their natural habitat in humans, patients, animals or the environment. This includes the different options of single growth and competitive growth experiments and modifications of growth conditions, which has not been undertaken here (nutrition depletion, anaerobic conditions, etc.). Second, we assessed the exponential growth rate as a measure to compare fitness, but we are well aware that also other endpoints such as time to reach stationary phase or maximum cell density are other possible markers. Third, one limitation corresponds to a finding of this study showing that identical experimental settings may lead to contrary results. It was demonstrated that *bla*_CMY-16_-mediated plasmid transfer may lead to a fitness gain or loss in doing the same experiment twice. Thus, fitness effects may be caused by side-effects associated with but potentially independent from the measured and selected (resistance) plasmid transfer. Lastly, we were unable to experimentally confirm a causal link between the measured fitness gain and the described genetic changes in the transconjugants’ genomes simply due to fact that the funding for this research project expired.

## 4. Materials and Methods

### 4.1. Strains

Altogether, 43 strains of *E. coli* (n = 38), *K. pneumoniae* (n = 3), *K. oxytoca* (n = 1) and *E. cloacae* (n = 1) were selected as donors from previous studies [19,20,22,23] and from routine diagnostics sent for strain typing and beta-lactamase characterization to our laboratory (Supplementary Table S1). Majority of the selected donor strains (n = 26) were *E. coli* ST131 with *bla*_CTX-M-15_, while others were *E. coli* strains possessing other *bla*_CTX-M_ variants (*bla*_CTX-M-1_, *bla*_CTX-M-14_), *E. coli* strains possessing *bla*_CMY_ variants (*bla*_CMY-2_ and *bla*_CMY-16_) and *E. coli* strains possessing various carbapenemase genes (*bla*_OXA-48_, *bla*_VIM-1_, *bla*_NDM-1_). For mating experiments, a sodium azide-resistant (Azi^r^) recipient strain *E. coli* J53 was used [55].

### 4.2. Transfer Experiments and Characterization of Transconjugants

Transfer of the beta-lactamase gene-carrying plasmids was conducted using broth mating and recipient strain *E. coli* J53 Azi^r^. Transconjugants were cultivated on Luria-Bertani (LB) agar with cefotaxime (CTX, 1 mg/L), cefoxtin (CXI, 10 mg/L), ampicillin (AMP, 50 mg/L) and 200 mg/L sodium azide, morphologically checked and screened by PCR for presence of the respective beta-lactamase genes (Supplementary Tables S1 and S6). Furthermore, the plasmid DNA of the transconjugants was extracted and the replicon types of the transferred plasmids were characterized using the PBRT Kit 2.0 (Diatheva, Fano PU, Italy). The plasmid sizes were determined by S1-nuclease restriction and pulsed-field gel electrophoresis [56].

### 4.3. Growth Experiments in Microtiter Plates

An overnight culture was adjusted to 5 × 10^6^ colony forming units (CFU)/mL in fresh LB-medium at 37 °C. Growth was measured in parallel in the Bioscreen C MBR photometer in a microtiter volume (Oy Growth Curves Ab Ltd., Helsinki, Finland). For this purpose, 200 μL of the cell suspension was pipetted in triplicate into the wells of a 96-well honeycomb plate (Oy Growth Curves Ab Ltd. (formerly: Bioscreen), Helsinki, Finland) and measured at 37 °C for 20 h. The optical density (OD_600_) was determined every 10 min. Before each OD measurement, the bacterial suspensions were mixed by short-vortexing. Reference strains were carried out on each plate to determine the relative fitness. The reference strains used were *E. coli* J53 Azi^r^, and for the long-term growth experiments, additionally, the transconjugants of the respective G_0_ generation. 

### 4.4. Long-Term Growth Experiments

Based on the initial fitness characterization, seven transconjugants with characteristic fitness effects (102/04 K1, 102/04 K2, 252/09 K2, 104/15 K3, RS165 K1, 151/09 K2, 346/12 K2) were identified, which were subsequently cultivated for 500 generations and investigated for possible fitness compensation effects. The initial inoculum of long-term cultivation experiments resulted from a single colony of a respective transconjugant, which was suspended into 5 mL LB medium and incubated at 37 °C in a shaking incubator at 140 rpm. After about 24 h, 10 μL of the bacterial suspension was transferred into 5 mL fresh LB medium and incubated again for 24 h. An inoculation volume of 10 μL resulted in an increase of 8.96 generations per passage (24 h). The cultivation was carried out over 500 generations. Every 100 generations, bacterial culture was sampled and cryopreserved at −80 °C. Cultures of isolates after 200 (G_200_) and 500 generations (G_500_) were examined for possible changes in their fitness and antimicrobial susceptibilities. For this purpose, the cell suspension was plated in a dilution of 10^−6^ each onto LB agar and LB agar containing selective antibiotics (CXI: 10 mg/L, AMP: 50 mg/L). From the antibiotic-containing agar plates, four colonies per each generation were selected and examined for their fitness properties by growth measurements, antibiotic susceptibility testing by broth microdilution for 13 antibiotics (AMP, CTA, CTZ, CXI, GEN, KAN, AMI, STR, NAL, CMP, CIP, MER, TRS; see EUCAST abbreviations) according to EUCAST (European Committee on Antimicrobial Susceptibility Testing) recommendations and the presence of beta-lactamase genes by PCR.

### 4.5. Determining Growth Characteristics and Relative Fitness

To determine the relative fitness, the growth rate in the exponential phase was used as a fitness parameter. For this purpose, the growth rates μ_tx_ (time x) were first determined for all consecutive measurement points using the following formula with OD_1_ at t_1_ and OD_2_ at t_2_:

(1) $$\mu_{tx}=\frac{InOD_{2}-InOD_{1}}{t_{2}-t_{1}}$$

The exponential growth rate was determined by an initial series of experiments with the plasmid-free recipient *E. coli* J53 Azi^r^. For the present experimental conditions, a threshold of μ_tx_ ≥ 0.5 (h^−1^) was defined as exponential growth. To exclude measurement errors or outliers outside the exponential growth phase, the additional condition was defined that μ_tx_ at t_x_ is only assigned to the exponential phase if μ_tx-1_ at t_x-1_ and μ_tx+1_ at t_x+1_ are also above the threshold. The growth rate μ for the entire exponential phase was then determined by the mean value of all growth rates, μ_tx_, that meet the conditions and are above the threshold. The respective triplicates of an isolate (= technical triplicate) were combined to a mean (average) value and the standard deviation was determined. Using the growth rate, μ, the relative fitness of each isolate compared to the recipient, transconjugants of the different generations or wildtype strains were calculated. The relative growth rate was determined by the growth rate of a transconjugant μ divided by the growth rate of the recipient *E. coli* J53 Azi^r^ (multiplied by 100%). The growth rates of *E. coli* J53 Azi^r^, the wildtype or transconjugant G_0_ isolates were used to determine reference fitness values. Deviations from these growth rates resulted in the relative fitness loss/gain in percent (%). All measurements were performed at least in biological triplicates, resulting in a mean (average) value (of the mean values of the technical triplicates).

### 4.6. Isolation of DNA, Plasmid DNA and Genomic DNA for Sequencing

Genomic DNA for PCR screening of resistance genes was isolated by simple heating and subsequent centrifugation. Genomic DNA for next-generation sequencing (NGS) was isolated with the DNeasy Blood and Tissue Kit (Qiagen GmbH, Hilden, Germany) using a standard protocol. Genomic DNA for single-molecule real-time (SMRT) sequencing was isolated with a Genomic-tip 100/G Kit (Qiagen GmbH). The quality, quantity and purity of the DNA were determined by agar gel electrophoresis, photometrically using a BioPhotometer (Eppendorf, Hamburg, Germany) and in a QuBit 4 fluorometer (Thermo Fisher Scientific, Inc., Waltham, MA, USA) with a Qubit dsDNA HS Assay Kit (Thermo Fisher Scientific, Inc.). Plasmid DNA was extracted by the Plasmid Mini Kit (Qiagen GmbH, Hilden, Germany). Sizes of plasmids were assessed after agarose gel electrophoresis compared to plasmids of known sizes using reference strains *E. coli* V517 and *E. coli* R222.

### 4.7. DNA Sequencing by NGS and SMRT Sequencing

For subsequent bioinformatic analyses, seven different 102/04 K1 (G_0_, G_500_-1) and K2 (G_0_) isolates as well as RS165 K1 (G_0_, G_200_-1, G_200_-3, G_500_-3) isolates of different generations were sequenced using Illumina whole-genome sequencing. For reference purposes, the G_0_ transconjugants of 102/04 K1 and 102/04 K2 were long-read sequenced using SMRT sequencing. Sequencing libraries were generated using the Nextera XT DNA Library Preparation Kits (Illumina, Inc., San Diego, CA, USA), as specified by the manufacturer. NGS sequencing was performed at the Core Facility of the Robert Koch Institute MF2, on a MiSeq system (Illumina, Inc.) using the MiSeq v3 Reagent Kit (Illumina, Inc.) with 2 × 300 bp paired-end mode. SMRT Sequencing was executed on a Pacific Biosciences apparatus RS II at GATC Biotech AG (now: MWG Eurofins, Konstanz, Germany). The sample preparation (DNA extraction) was performed according to the specifications of the service provider and as described above.

### 4.8. Whole-Genome Data Analysis

The quality of the generated Illumina raw data was checked using the program FastQC (https://github.com/s-andrews/FastQC). The quality of the raw data was improved by filtering out or shortening reads with poor-quality parameters. Trimmomatic was applied to filter and exclude raw reads of poor quality, if necessary [57]. For subseqeunt de novo assembly of raw data, the parameter “maxinfo 15:0.5” was chosen. All further parameters were used as default settings. The raw data had an average Phred value of more than 30 and the average read quality could be increased to a PHRED score of more than 35 after trimming. Illumina raw data were assembled using SPAdes (v.3.10.1) [58]. The PacBio whole-genome data (102-04 K1 G_0_, 102-04 K2 G_0_) were assembled by the service provider using the HGAP software (v.3), resulting in two contigs per sequencing: one contig covering the complete chromosome and one covering the plasmid. Polishing and ring closing of long-read data were conducted by aligning of trimmed Illumina read data (parameter “slidingwindow 4:15”) on the HGAP3-generated contigs using Unicycler [59]. The assignment of the contigs as chromosomal or plasmid sequence was done by a BLAST analysis. The protein coding sequence segments of the consensus sequences of chromosome and plasmid were annotated using RAST annotation servers [60].

The identification of sequence modifications of “later-generation/long-time cultivated” strains compared to the generation G_0_ strains, was conducted by using the long-read-based reconstructed G_0_ strains 102/04 K1 and 102/04 K2 as a reference. In detail, de novo assembled Illumina read-based contigs were aligned and orientated with the proprietary Geneious Mapper (Parameter: Medium–Low-Sensitivity, no iterations) on the reconstructed reference generation G_0_ genomes of 102/04 K1 and 102/04 K2 (Geneious v10.0.5 (Biomatters, Ltd., Auckland, New Zealand)) [61]. This procedure enabled a better identification of transpositions and insertions in the genome. Occurring insertions or variations from the reference were compared by *blastn* against the NCBI Genbank Nucleotide Collection (*nr*/*nt*). Mobile genetic elements, such as insertion sequences (IS), were identified and annotated in detail using the ISfinder database (https://isfinder.biotoul.fr) [62]. The final consensus sequence, created by using the reference-oriented contigs, including identified insertions and deletions, was then re-checked for sequence consistency by realigning the reads on the consensus. Finally, the sequences of the generation G_0_ and the long-time cultivated strains were compared using Mauve (http://darlinglab.org/mauve/mauve.html) [63]. The same procedure was used to reconstruct and compare the plasmid sequences. Annotations of the references were transferred on the newly reconstructed sequences. Resistance genes, plasmid replicon genes and selected insertion sequences were annotated using the PlasmidFinder 1.3, ResFinder 3.0 and ISfinder databases (http://www.genomicepidemiology.org/) [64,65].

## 5. Conclusions

Horizontal gene transfer appears frequently in nature and new “resistance gene–plasmid–host” combinations resulting from a single genetic event may arise regularly, and some of these new combinations may be fitter than their resistance plasmid-free progenitors. As the reservoir of transferable pAmpC-type resistance is a large problem in animal farming and in humans in some parts of the world, it cannot be excluded that a new variant appears once that successfully spreads across all sectors. This study showed the complexity of the resistance problem in terms of acquisition, fitness effects and biological consequences of horizontal resistance gene and mobile element transfer studied after an in vitro broth mating of beta-lactamase-, and especially *bla*_CMY_-carrying, plasmids transferred between Enterobacterales isolates. We showed that performing the same experiment twice could lead to opposite effects in terms of fitness burden or gain associated with an acquisition of a *bla*_CMY-16_-harboring plasmid in an *E. coli* host (Figure 1). We observed fitness compensation over hundreds of generations from both sides, starting from either a fitness loss or a fitness gain associated with resistance plasmid acquisition (Figure 2). Using a combination of long-read and short-read sequencing information, we were capable of identifying genomic changes appearing during growth of selected transconjugants for 500 generations in liquid culture (Figure 3 and Figure 4). However, when assessing and evaluating these genomic changes over time, we were unable to address direct and putatively causative links between measured fitness effects and insertions of mobile DNA or single nucleotide changes in the evolved transconjugants. We assume that the effects of these genomic changes might be indirect and regulatory, a hypothesis that requires further experimental proof in future experiments.

## Figures and Tables

**Table 1:** Characteristics of the transconjugants (Kx) used for experiments on bacterial fitness.

Isolate	Wildtype Host Species	Beta-Lactamase Gene	Plasmid Size (kb)	Replicon Type
RS039 K1	*E. coli*	*bla*_CTX-M-1_	80	IncI1
RS292 K1	*E. coli*	*bla*_CTX-M-15_, *bla*_TEM-1_	80	IncFII
17/11 K1	*E. coli*	*bla*_KPC-2_	190	IncA/C
370/12 K1	*K. oxytoca*	*bla*_VIM-1_	250	IncFIB
384/13 K2	*K. pneumoniae*	*bla*_NDM-1_, *bla*_CTX-M-15_, *bla*_OXA-9_, *bla*_TEM-1_	110	IncFII
656/13 K2	*E. coli*	*bla*_SHV-12_	100	IncI1
531/12 K2	*E. coli*	*bla*_CMY-2_	90	IncI1
252/09 K3 *	*E. coli*	*bla*_CMY-2_	85, 60	IncI1, IncFII
346/12 K2 *	*K. pneumoniae*	*bla*_OXA-48_	60	IncL/M-1
RS165 K1 *	*E. coli*	*bla*_CTX-M-14_	70	IncFII
151/09 K2 *	*E. cloacae*	*bla*_VIM-1_	100	IncN/IncR
104/15 K3 *	*E. coli*	*bla*_CTX-M-15_	77	IncF
102/04 K1 *	*E. coli*	*bla*_CMY-16_, *bla*_TEM-1_	160	IncA/C
102/04 K2 *	*E. coli*	*bla*_CMY-16_, *bla*_TEM-1_	160	IncA/C

* These isolates were selected for further experiments.

## Data Availability

All genome data are accessible via project number PRJEB41562 (https://www.ebi.ac.uk/ena/browser/home).

## Supplementary Materials

The following are available online at https://www.mdpi.com/2079-6382/10/1/90/s1, Table S1: Characteristics of clinical donor strains and the corresponding transconjugants used for fitness experiments, Table S2: Fitness values of the evolved transconjugants from long-term growth experiments, Table S3: Antibiotic susceptibilities (MICs in mg/L) of transconjugants of the different generations, detected by broth microdilution, Table S4: Results of de novo assembly of the reads of isolates 102/04 K1 G_0_ und K2 G_0_, Table S5: Results of read mapping of illumina data for evolved transconjugants of RS165 K1 onto the reference sequence of 102/04 K1 G_0_, Table S6: PCR primer pairs used in this study, Figure S1: Growth curves of seven selected transconjugants with beta-lactamase gene-carrying plasmids vs. the plasmid-free recipient *E. coli* J53 Azi^r^.
