# Supplementary material for: Genome-Based Analyses of Fitness Effects and Compensatory Changes Associated with Acquisition of blaCMY-, blaCTX-M-, and blaOXA-48/VIM-1-Containing Plasmids in Escherichia coli
Source: Antibiotics (Basel). 2021 Jan 19;10(1):90. doi: 10.3390/antibiotics10010090 (PMC7832316; doi:10.3390/antibiotics10010090)
Supplement: Supplementary file 1 [file antibiotics-10-00090-s001.pdf]

Pietsch et al., Supplementary Files

Supplementary Table S1: Characteristics of clinical donor strains and the corresponding transconjugants (Kx)used for fitness experiments.

Isolate	Transconjugant[1]	Species	Year of isolation	MLST/ Phylogenetic group[2]	*Beta-lactamase* gene	*bla* plasmid [kb][3]	Replicon type[4]
102/04		*E. coli*	2004	D	bla_{CMY-16}, bla_{TEM-1}	160	
	102/04 K1	*E. coli*		A	bla_{CMY-16}, bla_{TEM-1}	160	IncA/C
	102/04 K2	*E. coli*		A	bla_{CMY-16}, bla_{TEM-1}	160	IncA/C
151/09		*E. cloacae*	2009		bla_{VIM-1}	100	
	151/09 K2	*E. coli*		A		100	IncN/IncR
17/11		*E. coli*	2011	B1	bla_{KPC-2}	210, 190, 85, 65	
	17/11 K1	*E. coli*		A	bla_{KPC-2}	190	IncA/C
252/09		*E. coli*	2009		bla_{CMY-2}, bla_{TEM-1}	85, 60, 30	IncI1,IncFII
	252/09 K3	*E. coli*		A	bla_{CMY-2}	85, 60	IncI1, IncFII
346/12		*K. pneumoniae*	2012		bla_{OXA-48}, bla_{OXA-1}, bla_{OXA-9}	105, 60, 35	IncL/M-1
	346/12 K2	*E. coli*		A	bla_{OXA-48}	60	IncL/M-1
370/12		*K. oxytoca*	2012		bla_{VIM-1}, bla_{OXA},	250, 150, 100	IncFIB, IncL/M-1
	370/12 K2	*E. coli*		A	bla_{VIM-1}	250	IncFIB
384/13		*K. pneumoniae*	2013		bla_{NDM-1}, $bla_{SHV-like}$, $bla_{CTX-M-15}$, $bla_{OXA-232}$, bla_{OXA-1}, bla_{OXA-9}, bla_{TEM}	240, 110	IncFII, IncI1, IncN?
	384/13 K2	*E. coli*		A	bla_{NDM}, $bla_{CTX-M-15}$, bla_{OXA-9}, bla_{TEM-1}	110	IncFII
467/13		*K. pneumoniae*			bla_{OXA-48}	60	IncL/M-1
	467/13 K2	*E. coli*		A	bla_{OXA-48}	60	
531/12		*E. coli*			bla_{CMY-2}	190, 90, 55	IncI1
	531/12 K2	*E. coli*		A		90	IncI1
656/13		*E. coli*			bla_{SHV-12}	140, 105	
	656/13 K2	*E. coli*		A	bla_{SHV-12}	105	IncI1
RS014	*negative*	*E. coli*	2010	ST131	$bla_{CTX-M-15}$		
RS039		*E. coli*	2011	ST46	$bla_{CTX-M-1}$, bla_{TEM-1}	80	IncI1
	RS039 K1	*E. coli*		A	$bla_{CTX-M-1}$		IncI1
RS060	*negative*	*E. coli*	2011	ST131	$bla_{CTX-M-27}$		
RS135	*negative*	*E. coli*	2011	ST410	$bla_{CTX-M-15}$		
RS081	*negative*	*E. coli*	2011	D	$bla_{CTX-M-1}$, bla_{TEM-1}	90	IncI1
RS165		*E. coli*	2011	ST73	$bla_{CTX-M-14}$		
	RS165 K1				$bla_{CTX-M-14}$	70	IncFII
RS292		*E. coli*	2012	D	$bla_{CTX-M-15}$, bla_{TEM-1}		IncFII
	RS292 K1	*E. coli*		A	$bla_{CTX-M-15}$, bla_{TEM-1}		IncFII
104/15		*E. coli*	2015	B2, rfbO25 negative	$bla_{CTX-M-15}$	77	IncF

Isolate	Transconjugant[1]	Species	Year of isolation	MLST/ Phylogenetic group[2]	*Beta-lactamase* gene	*bla* plasmid [kb][3]	Replicon type[4]
	104/15 K3	*E. coli*		A	$bla_{CTX-M-15}$	77	IncFII
RS013	*negative*	*E. coli*	2010	ST131	$bla_{CTX-M-15}$		
RS014	*negative*	*E. coli*	2010	ST131	$bla_{CTX-M-15}$		
RS025	*negative*	*E. coli*	2011	ST131	$bla_{CTX-M-15}$		
RS029	*negative*	*E. coli*	2011	ST131	$bla_{CTX-M-15}$		
RS031	*negative*	*E. coli*	2011	ST131	$bla_{CTX-M-15}$		
RS050	*negative*	*E. coli*	2011	ST131	$bla_{CTX-M-15}$		
RS062	*negative*	*E. coli*	2011	ST131	$bla_{CTX-M-15}$		
RS071	*negative*	*E. coli*	2011	ST131	$bla_{CTX-M-15}$		
RS072	*negative*	*E. coli*	2011	ST131	$bla_{CTX-M-15}$		
RS080	*negative*	*E. coli*	2011	ST131	$bla_{CTX-M-15}$		
RS090	*negative*	*E. coli*	2011	ST131	$bla_{CTX-M-15}$		
RS093	*negative*	*E. coli*	2011	ST131	$bla_{CTX-M-15}$		
RS098	*negative*	*E. coli*	2011	ST131	$bla_{CTX-M-15}$		
RS099	*negative*	*E. coli*	2011	ST131	$bla_{CTX-M-15}$		
RS103	*negative*	*E. coli*	2011	ST131	$bla_{CTX-M-15}$		
RS104	*negative*	*E. coli*	2011	ST131	$bla_{CTX-M-15}$		
RS106	*negative*	*E. coli*	2011	ST131	$bla_{CTX-M-15}$		
RS107	*negative*	*E. coli*	2011	ST131	$bla_{CTX-M-15}$		
RS113	*negative*	*E. coli*	2011	ST131	$bla_{CTX-M-15}$		
RS115	*negative*	*E. coli*	2011	ST131	$bla_{CTX-M-15}$		
RS127	*negative*	*E. coli*	2011	ST131	$bla_{CTX-M-15}$		
RS134	*negative*	*E. coli*	2011	ST131	$bla_{CTX-M-15}$		
RS137	*negative*	*E. coli*	2011	ST131	$bla_{CTX-M-15}$		
RS143	*negative*	*E. coli*	2011	ST131	$bla_{CTX-M-15}$, bla_{TEM-1}		
RS184	*negative*	*E. coli*	2011	ST131	$bla_{CTX-M-15}$		

Legend: [1] recipient *E. coli* K J53 Azi^r was used in mating experiments, [2] PCR-based determination of *E. coli* phylogenetic group (Clermont O, Bonacorsi S, Bingen E. Rapid and simple determination of the *Escherichia coli* phylogenetic group. Appl Environ Microbiol. 2000;66(8):4555) and determination of sequence type (ST) by multilocus sequence typing (https://pubmlst.org/bigsdb?db=pubmlst_ecoli_achtman_seqdef), [3] sizes of plasmids that contained the beta-lactamase (*bla*) gene of interest (printed in bold) were determined by S1 nuclease treatment and subsequent PFGE analyses, [4] PCR based replicon typing using the PRBT kit (Diatheva, Cartoceto, Italy).

Supplementary Table S2: Fitness values of the initial and evolved transconjugants from long-term growth experiments. Fitness values of the initial transconjugants relative to the fitness of the recipient *E. coli* J53 Azi^{r} [(1)] is given as generation G_0, values of the evolved transconjugants are presented for generations G_0, G_{200}, and G_{500}. The numbers of repeated experiments (*n* attempts) and the standard deviations (SD) are given.

**Isolate**	**Generation G_0 relative fitness [%]** Average value (*n* attempts)	SD	**Generation G_{200} relative fitness [%]** Average value (*n* attempts)	SD	**Generation G_{500} relative fitness [%]** Average value (*n* attempts)	SD
**J53[1]**	100					
**J53-1**			102.03 (2)	0.36	96.87 (3)	1.86
**J53-2**			101.31 (2)	0.31	93.54 (3)	1.31
**J53-3**			bdl (2)		94.68 (3)	1.78
**J53-4**			102.81 (2)	2.77	97.41 (3)	2.20
**RS165K1**	98.47 (8)	1.76				
**RS165 K1-1**			113.19 (2)	2.85	99.08 (3)	1.12
**RS165 K1-2**			bdl (2)		99.95 (3)	0.55
**RS165 K1-3**			101.29 (2)	0.83	99.11 (3)	3.25
**RS165 K1-4**			112.63 (2)	3.18	97.66 (3)	2.18
**102/04 K1**	107.23 (4)	2.29				
**102/04 K1-1**			110.38 (2)	1.09	99.04 (3)	1.49
**102/04 K1-2**			96.16 (2)	0.65	102.68 (3	3.63
**102/04 K1-3**			99.46 (1)		98.89 (3)	1.78
**102/04 K1-4**			100.49 (1)		102.10 (3)	2.33
**102/04 K2**	95.58 (3)	0.71				
**102/04 K2-1**			96.81 (2)	0.19	88.07 (3)	3.24
**102/04 K2-2**			97.99 (2)	0.64	104.53 (3)	0.57
**102/04 K2-3**			101.66 (1)		102.09 (3)	1.42
**102/04 K2-4**			99.89 (1)		94.75 (3)	0.60
**252/09 K3**	98.70 (3)	1.45				
**252/09 K3-1**			101.68 (1)		92.69 (3)	1.07
**252/09 K3-2**			101.39 (1)		95.65 (3)	2.12
**252/09 K3-3**			103.23 (1)		93.49 (3)	0.35
**252/09 K3-4**			93.57 (1)		97.86 (3)	2.02
**151/09 K2**	96.30 (3)	0.76				
**151/09 K2-1**			89.81 (1)		100.37 (3)	1.42
**151/09 K2-2**			93.74 (1)		97.60 (3)	0.58
**151/09 K2-3**			95.21 (1)		92.42 (3)	0.65
**151/09 K2-4**			90.82 (1)		95.59 (3)	1.36
**104/15 K3**	100.74 (3)	0.25				
**104/15 K3-1**			102.09 (2)	1.21	99.16 (3)	1.46
**104/15 K3-2**			101.26 (2)	1.51	97.99 (3)	1.40
**104/15 K3-3**			100.01 (2)	1.80	99.58 (3)	2.38
**104/15 K3-4**			100.70 (2)	1.81	95.20 (3)	1.93
**346/12 K2**	97.88 (3)	0.84				
**346/12 K2-1**			96.17 (1)		87.19 (3)	1.70
**346/12 K2-2**			97.60 (1)		83.70 (3)	0.60
**346/12 K2-3**			95.86 (1)		84.05 (2)	1.36
**346/12 K2-4**			95.50 (1)		88.07 (3)	1.40
**RS039 K1***	99.02 (8)	2.15				
**RS292K1***	100.02 (8)	1.93				
**17/11K1***	99.76 (4)	1.52				
**370/12K1***	100.82 (5)	1.46				
**384/13K2***	100.01 (4)	2.33				
**656/13 K2***	98.47 (3)	0.84				
**531/12 K2***	98.34 (3)	0.16				

Legend: bdl = below detection limit; *isolates not selected for further analyses

Supplementary Table S3: Antibiotic susceptibilities (MICs in mg/L) of transconjugants of the different generations, detected by broth microdilution (EUCAST v10.0; https://www.eucast.org/clinical_breakpoints/).

Isolate	AMP	CTA	CTZ	CXI[1]	GEN	KAN[1]	AMI	STR[1]	NAL[1]	CMP	CIP	MER	TRS
**102/04 K1**	>16	>16	>32	>32	1	>32	>32	>64	16	>32	≤0.063	≤0.063	>128
**G200 -1**	>16	16	32	>32	≤0.5	>32	16	64	8	>32	≤0.063	≤0.063	>128
**G200 -2**	>16	8	16	16	≤0.5	>32	8	16	16	>32	≤0.063	≤0.063	>128
**G200 -5**	>16	>16	>32	>32	4	>32	>32	>64	16	>32	≤0.063	≤0.063	>128
**G200 -8**	>16	>16	>32	>32	≤0.5	>32	32	64	16	>32	≤0.063	≤0.063	>128
**G500 -1**	>16	8	16	16	≤0.5	>32	4	16	16	>32	≤0.063	≤0.063	>128
**G500 -2**	>16	16	>32	>32	≤0.5	>32	8	16	8	>32	≤0.063	≤0.063	> 128
**G500 -3**	>16	8	16	32	≤0.5	>32	8	16	16	>32	≤0.063	≤0.063	>128
**G500 -4**	>16	8	32	>32	≤0.5	>32	8	64	16	>32	≤0.063	≤0.063	>128
**102/04 K2**	>16	>16	>32	>32	≤0.5	>32	>32	>64	8	>32	≤0.063	≤0.063	>128
**G200 -1**	>16	>16	>32	>32	≤0.5	>32	>32	>64	<4	>32	≤0.063	≤0.063	>128
**G200 -2**	>16	>16	>32	>32	≤0.5	>32	>32	>64	<4	>32	≤0.063	≤0.063	>128
**G200 -5**	>16	>16	>32	>32	≤0.5	>32	>32	64	<4	>32	≤0.063	≤0.063	>128
**G200 -8**	>16	>16	>32	>32	≤0.5	>32	>32	>64	<4	>32	≤0.063	≤0.063	>128
**G500 -1**	>16	4	4	32	≤0.5	>32	4	8	<4	>32	≤0.063	≤0.063	>128
**G500 -2**	>16	>16	>32	>32	≤0.5	>32	>32	>64	<4	>32	≤0.063	≤0.063	>128
**G500 -3**	>16	>16	>32	>32	≤0.5	>32	>32	>64	<4	>32	≤0.063	≤0.063	>128
**G500 -4**	>16	16	32	>32	≤0.5	>32	16	32	<4	>32	≤0.063	≤0.063	>128
**252/09 K3**	> 16	16	>32	>32	≤0.5	≤2	≤2	≤4	8	8	≤0.063	≤0.063	≤4
**G200 -1**	>16	16	>32	>32	4	4	4	8	16	8	≤0.063	≤0.063	≤4
**G200 -2**	>16	16	>32	>32	1	4	4	8	16	8	≤0.063	≤0.063	≤4
**G200 -3**	>16	16	32	>32	1	4	4	≤4	8	8	≤0.063	≤0.063	≤4
**G200 -4**	>16	4	8	>32	2	≤2	4	8	16	8	≤0.063	≤0.063	≤4
**G500 -1**	>16	2	8	32	≤0.5	≤2	≤2	≤4	8	8	≤0.063	≤0.063	≤4
**G500 -2**	>16	4	8	16	≤0.5	≤2	≤2	≤4	8	8	≤0.063	≤0.063	≤4
**G500 -3**	>16	2	16	32	≤0.5	≤2	≤2	≤4	8	8	≤0.063	≤0.063	≤4
**G500 -4**	>16	16	>32	>32	4	8	4	8	16	8	≤0.063	≤0.063	≤4
**104/15 K3**	>16	>16	32	16	8	8	4	8	8	>32	≤0.063	≤0.063	≤4
**G200 -1**	>16	>16	32	16	>8	16	<2	<4	16	>32	≤0.063	≤0.063	≤4
**G200 -2**	>16	>16	32	8	>8	8	8	8	16	>32	≤0.063	≤0.063	≤4
**G200 -3**	>16	>16	32	8	>8	8	4	<4	16	>32	≤0.063	≤0.063	≤4
**G200 -4**	>16	>16	>32	32	>8	8	<2	<4	16	>32	≤0.063	≤0.063	≤4
**G500 -1**	>16	>16	32	16	>8	4	≤2	≤4	8	>32	≤0.063	≤0.063	≤4
**G500 -2**	>16	>16	>32	8	>8	8	4	≤4	8	>32	≤0.063	≤0.063	≤4
**G500 -3**	>16	>16	32	8	>8	16	≤2	≤4	16	>32	≤0.063	≤0.063	≤4
**G500 -4**	>16	>16	32	8	>8	8	≤2	≤4	16	>32	≤0.063	≤0.063	≤4

**Isolate**	**AMP**	**CTA**	**CTZ**	**CXI**[1]	**GEN**	**KAN**[1]	**AMI**	**STR**[1]	**NAL**[1]	**CMP**	**CIP**	**MER**	**TRS**
**RS165 K1**	>16	>16	≤2	2–16	≤0.5	≤2	≤2	≤4	8	8	≤0.063	≤0.063	≤4
**G200 -1**	>16	16	≤2	4	≤0.5	≤2	≤2	≤4	8	8	≤0.063	≤0.063	≤4
**G200 -2**	>16	8	≤2	8	≤0.5	≤2	≤2	≤4	8	8	≤0.063	≤0.063	≤4
**G200 -3**	>16	16	≤2	4	≤0.5	≤2	≤2	≤4	8	8	≤0.063	≤0.063	≤4
**G200 -4**	>16	16	≤2	4	≤0.5	≤2	≤2	≤4	16	8	≤0.063	≤0.063	≤4
**G500 -1**	>16	>16	<2	8	≤0.5	4	4	<4	8	8	≤0.063	≤0.063	≤4
**G500 -2**	>16	>16	4	4	≤0.5	<2	<2	<4	16	8	≤0.063	≤0.063	≤4
**G500 -3**	>16	>16	<2	8	2	4	<2	8	8	8	≤0.063	≤0.063	≤4
**G500 -4**	>16	>16	<2	8	2	4	4	<4	8	8	≤0.063	≤0.063	≤4
**151/09 K2**	>16	>16	>32	>32	4	>32	>32	>64	8	8	≤0.063	1	>128
**G200 -1**	>16	>16	>32	>32	1	32	32	>64	16	8	≤0.063	2	>128
**G200 -2**	>16	>16	>32	>32	4	32	16	>64	16	8	≤0.063	4	>128
**G200 -3**	>16	>16	>32	>32	8	32	>32	>64	16	8	≤0.063	4	>128
**G200 -4**	>16	>16	>32	>32	4	32	32	>64	16	8	≤0.063	4	>128
**G500 -1**	>16	>16	>32	>32	1	>32	4	>64	8	8	≤0.063	0.5	>128
**G500 -2**	>16	>16	>32	>32	<0.5	>32	4	4	8	8	≤0.063	0.25	>128
**G500 -3**	>16	>16	32	>32	<0.5	>32	<2	16	<4	<4	≤0.063	0.125	>128
**G500 -4**	>16	>16	32	>32	1	>32	16	>64	16	8	≤0.063	1	>128
**346/12 K2**	>16	≤1	≤2	2–16	≤0.5	4	≤2	8	8	8	≤0.063	0.25	≤4
**G200 -1**	>16	≤1	≤2	4	1	≤2	≤2	≤4	8	8	≤0.063	≤0.063	≤4
**G200 -2**	>16	<1	<2	8	≤0.5	<2	≤2	<4	8	8	≤0.063	0.125	≤4
**G200 -3**	>16	<1	<2	4	≤0.5	<2	≤2	<4	16	8	≤0.063	0.25	≤4
**G200 -4**	>16	<1	<2	4	≤0.5	2	≤2	<4	16	8	≤0.063	0.25	≤4
**G500 -1**	>16	<1	<2	4	≤0.5	<2	≤2	<4	8	8	≤0.063	0.25	≤4
**G500 -2**	>16	<1	<2	8	≤0.5	<2	≤2	<4	16	8	≤0.063	0.125	≤4
**G500 -3**	>16	<1	<2	4	≤0.5	<2	≤2	<4	8	8	≤0.063	0.125	<4
**G500 -4**	>16	<1	<2	8	<0.5	<2	<2	<4	8	8	≤0.063	≤0.063	<4

Legend: All abbreviations according to EUCAST[1] for these substances only ECOFF values are available (https://www.eucast.org/mic_distributions_and_ecoffs/)-https://www.eucast.org/fileadmin/src/media/PDFs/EUCAST_files/Disk_test_documents/Disk_abbreviations/EUCAST_system_for_antimicrobial_abbreviations.pdf

Supplementary Table S4: Results of *de novo* Assembly of the reads of isolates 102/04 K1 G_0 und K2 G_0.

Heading	**102/04K1**[1]	**p102/04K1**[1]	**102/04K2**[1]	**p102/04K2**[1]	**102/04K1 G_{500}-1**	**p102/04K1 G_{500}-1**
**Size**	4,687,587	164,040	4,685,473	164,040	4,690,582	165,381
**GC content**	50.8 %	52.3 %	50.8 %	52.3 %	50.8 %	52.8 %
**CDS**	4,495	206	4,493	206	4,498	207
**rRNA**	22	-	22	-	22	-
**tRNA**	86	-	86	-	86	-

Legend: [1] generation G_0.

Supplementary Table S5: Results of read mapping of illumina data for evolved transconjugants of RS165 K1 onto the reference sequence of 102/04 K1 G_0.

Heading	**RS165K1 G_0**	**pRS165K1 G_0**	**RS165K1 G_{200}-1**	**pRS165K1 G_{200}-1**	**RS165K1 G_{200}-3**	**pRS165K1 G_{200}-3**	**RS165K1 G_{500}-3**	**pRS165K1 G_{500}-3**
**Size**	4,682,662	70,269	4,686,841	70,269	4,687,417	70,269	4,688,104	70,269
**GC content**	50.8 %	52.3 %	50.8 %	52.3 %	50.8 %	52.3 %	50.8 %	52.3 %
**CDS**	4,508	96	4,511	96	4,512	96	4,512	96
**rRNA**	22	-	22	-	22	-	22	-
**tRNA**	86	-	86	-	86	-	86	-

Supplementary Table S6: Used PCR primer pairs in this study.

Primer	T_a [°C]	nM Multiplex	Product size [bp]	Sequence 5´-->3´	Reference
blaVIM fwd<br>blaVIM rev	55		216	AGTGGTGAGTATCCGACAG<br>ATGAAAGTGCGTGGAGAC	1
ChuA fwd<br>ChuA rev	55	200	279	GACGAACCAACGGTCAGGAT<br>TGCCGCCAGTACCAAAGACA	2
CITM fwd<br>CITM rev	60		462	TGGCCAGAACTGACAGGCAAA<br>TTTCTCCTGAACGTGGCTGGC	3
CTX-M universal fwd<br>CTX-M universal rev	55	267	551	CGCTTTGCGATGTGCAG<br>ACCGCGATATCGTTGGT	5
CTX-M-9 MP fwd<br>CTX-M-9 MP rev	55	200	356	GCAGTACAGCGACAATACCG<br>TATCATTGGTGGTGCCGTAG	5
gndbis fwd<br>rfbO25b rev	59		300	ATACCGACGACGCCGATCTG<br>TGCTATTCATTATGCGCAGC	6
gndbis fwd<br>rfbO16 rev	59		450	ATACCGACGACGCCGATCTG<br>GGATCATTTATGCTGGTACG	6
KPCu fwd<br>KPCu rev	58		533	CAGCTCATTCAAGGGCTTTC<br>AGTCATTTGCCGTGCCATAC	5
OXA-48 fwd<br>OXA-48 rev	58		555	AAATCACAGGGCGTAGTTGTG<br>GACCCACCAGCCAATCTTAG	5
SHV MP1 fwd<br>SHV MP1 rev	55	267	750	TTCGCCTGTGTATTATCTCC<br>TCCGCTCTGCTTTGTTATTC	5
TEM universal fwd<br>TEM universal rev	55	535	851	ATGAGTATTCAACATTTCCG<br>TTAATCAGTGAGGCACCTAT	5
TspE4C fwd<br>TspE4C rev	55	267	152	GAGTAATGTCGGGGCATTCA<br>CGCGCCAACAAAGTATTACG	2
Yja fwd<br>Yja rev	55	400	211	TGAAGTGTCAGGAGACGCTG<br>ATGGAGAATGCGTTCCTCAAC	2

References: (1) Miriagou V, Tzelepi E, Gianneli D, Tzouvelekis LS. Escherichia coli with a self-transferable, multiresistant plasmid coding for metallo-beta-lactamase VIM-1. Antimicrob Agents Chemother. 2003;47(1):395-7; (2) Clermont O, Bonacorsi S, Bingen E. Rapid and simple determination of the Escherichia coli phylogenetic group. Appl Environ Microbiol. 2000;66(8):4555; (3) Perez-Perez FJ, Hanson ND. Detection of plasmid-mediated AmpC beta-lactamase genes in clinical isolates by using multiplex PCR. J Clin Microbiol. 2002;40(6):2153-62; (5) Grobner S, Linke D, Schutz W, Fladerer C, Madlung J, Autenrieth IB, et al. Emergence of carbapenem-non-susceptible extended-spectrum beta-lactamase-producing Klebsiella pneumoniae isolates at the university hospital of Tubingen, Germany. J Med Microbiol. 2009;58(Pt 7):912-22; (6) Johnson JR, Clermont O, Johnston B, Clabots C, Tchesnokova V, Sokurenko E, et al. Rapid and specific detection, molecular epidemiology, and experimental virulence of the O16 subgroup within *Escherichia coli* sequence type 131. J Clin Microbiol. 2014;52(5):1358-65.

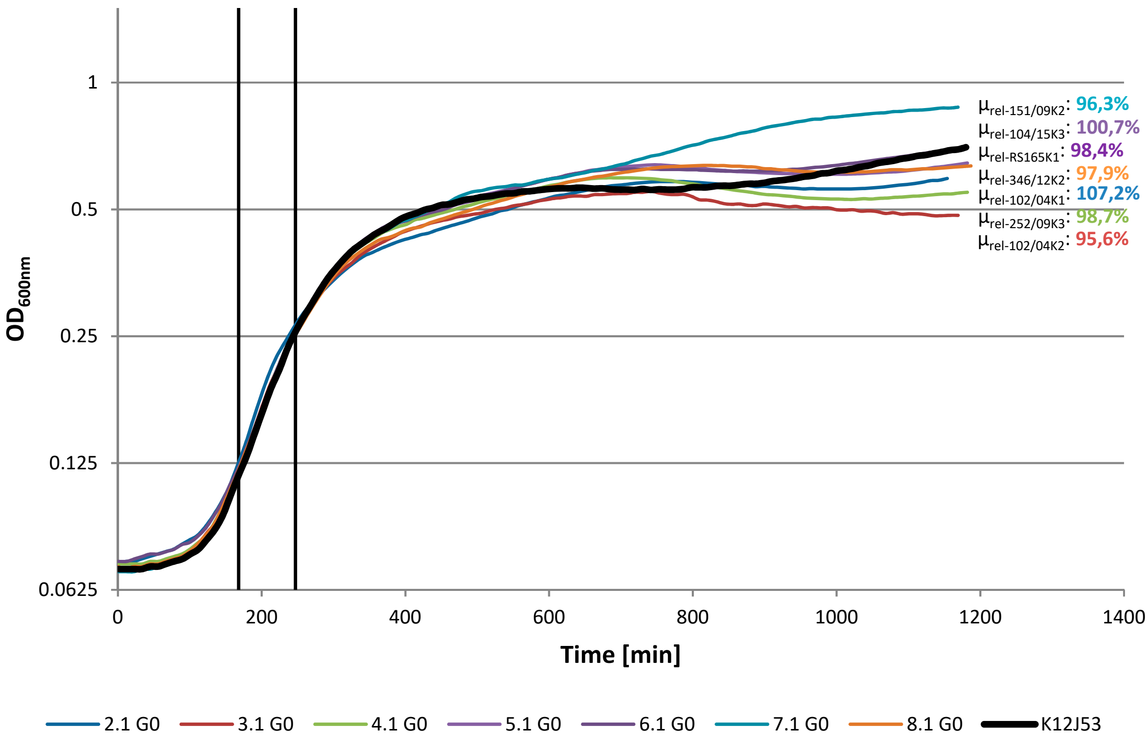

Figure S1. Growth curves of seven selected transconjugants with beta-lactamase gene carrying plasmids vs. the plasmid-free recipient *E. coli* J53 Azi^r. The black vertical lines frame the area of exponential growth used to determine the growth rates.
